# Personalized endoprostheses for the proximal humerus and scapulohumeral joint in dogs: Biomechanical study of the muscles’ contributions during locomotion

**DOI:** 10.1371/journal.pone.0262863

**Published:** 2022-01-24

**Authors:** Linh-Aurore Le Bras, Anatolie Timercan, Marie Llido, Yvan Petit, Bernard Seguin, Bertrand Lussier, Vladimir Brailovski

**Affiliations:** 1 Department of Mechanical Engineering, Ecole de Technologie Supérieure, Montréal, Québec, Canada; 2 Faculté de Médecine Vétérinaire, Département de Sciences Cliniques, Université de Montréal, Montréal, Québec, Canada; 3 Department of Clinical Sciences, College of Veterinary Medicine and Biomedical Sciences, Fort Collins, Colorado, United States of America; New York Institute of Technology, UNITED STATES

## Abstract

Osteosarcoma represents one of the most common bone tumours in dogs. It commonly occurs in the proximal humerus, the most affected anatomic site. Until recently, amputation or limb-sparing surgery leading to an arthrodesis coupled with chemotherapy were the only available treatments, but they often lead to complications, reduced mobility and highly impact dog’s quality of life. Prototypes of both articulated and monobloc (no mobility) patient-specific endoprostheses have been designed to spare the limb afflicted with osteosarcoma of the proximal humerus. This study focuses on the biomechanical effects of endoprostheses and shoulder muscle kinematics. For each of the endoprosthesis designs, a minimal number of muscles needed to ensure stability and a certain degree of joint movement during walking is sought. A quasi-static study based on an optimization method, the minimization of the sum of maximal muscle stresses, was carried out to assess the contribution of each muscle to the shoulder function. The identification of the most important muscles and their impact on the kinematics of the prosthetic joint lead to an improvement of the endoprosthesis design relevance and implantation feasibility.

## Introduction

Osteosarcoma is the most common bone tumor in dogs [1], especially large breed animals (Doberman, Golden retriever, Rottweiler). The dog’s age also impacts the prevalence of this disease, with older dogs facing a higher probability of developing osteosarcoma. The most affected anatomic site is the proximal humerus [1]. Currently, amputation of the affected limb is the most recommended therapeutic and palliative treatment option. However, this option is not optimal since the dog’s gait is subsequently impacted [2] and some dogs are not good candidates for amputation due to concomitant orthopedic diseases or neurologic conditions. Furthermore, amputation can also lead to some negative sequelae: metastasis, oedema, dehiscence of the wound or arthrosis of the ipsilateral limb and pneumonia [3,4]. Other reported treatment alternatives include intraoperative radiation therapy, stereotactic radiation therapy (SRT), or surgical limb-sparing using an allograft. Intraoperative radiotherapy, reported in 5 dogs with proximal humeral osteosarcoma, led to complications in all dogs, including bone fracture, implant failure, infection, and radial nerve paralysis [5]. The most common major complication with SRT is fracture. An estimated 62% of dogs had a fracture at 9 months after the radiation treatments [6]. The use of an allograft for the proximal humerus site has led to high complication rates and poor limb functions [7]. There are therefore currently no options for preserving the limb for proximal humeral osteosarcoma with an acceptable risk of complications.

As an alternative, the successful use of 3D-printed patient-specific endoprostheses (henceforth simplified as prostheses) has been recently reported in dogs afflicted by tumors of the distal radius, the second most affected by osteosarcoma anatomic site after the proximal humerus [8]. On the other hand, to date, only one shoulder joint hemiarthroplasty with an articulated prosthesis has been reported [9]. It was found that 105 weeks following this surgery, a postoperative analysis demonstrated that the affected limb never showed vertical force values similar to those in the healthy contralateral limb. That study concluded that further investigations are necessary to guarantee long-term outcome. To the best of the authors’ knowledge, there is a dearth of information on patient-specific prostheses in dogs afflicted with osteosarcoma of the proximal humerus.

After a prosthesis is surgically implanted in the canine shoulder, it must sustain forces exerted by the muscles attached. Studies carried out on the fixation of soft tissues on metallic prostheses with autogenous cancellous bone grafts have shown convincing results: after 6 to 12 weeks of recovery, muscles attached to such prostheses generate forces similar to muscles that were left intact [10–13]. However, the number of muscles required to be attached to canine shoulder prostheses and the forces they exert on them are currently still unknown.

There have been efforts to reference and characterize the muscles in the thoracic [14] and pelvic [15] limbs in dogs in order to define their insertion points on the bones as well as their morphometric data, which for each muscle include its mass, angle of pennation, length and cross-sectional area. Furthermore, muscular force contributions expressed as percentages of body weight were evaluated for the pelvic limbs based on these data [16], but not for their thoracic equivalents. The aim of this study is to use the morphometric data of thoracic limb muscles to determine their force contributions.

This study also aims to calculate the individual contributions of the muscles of the forelimb that have an impact on the scapulohumeral joint in three walking cycle positions, and to apply these data for the cases of articulated or monobloc prostheses. Understanding how these muscles contribute to the stability and function of the shoulder is necessary to help the surgeons identifying which muscles need to be preserved as salvaged during prosthesis implantation. It will also constitute requisite information for engineers for optimizing a personalized prosthesis design and ensure its biomechanical compatibility with the shoulder joint function.

## Limb-sparing technology description

**Fig 1:**
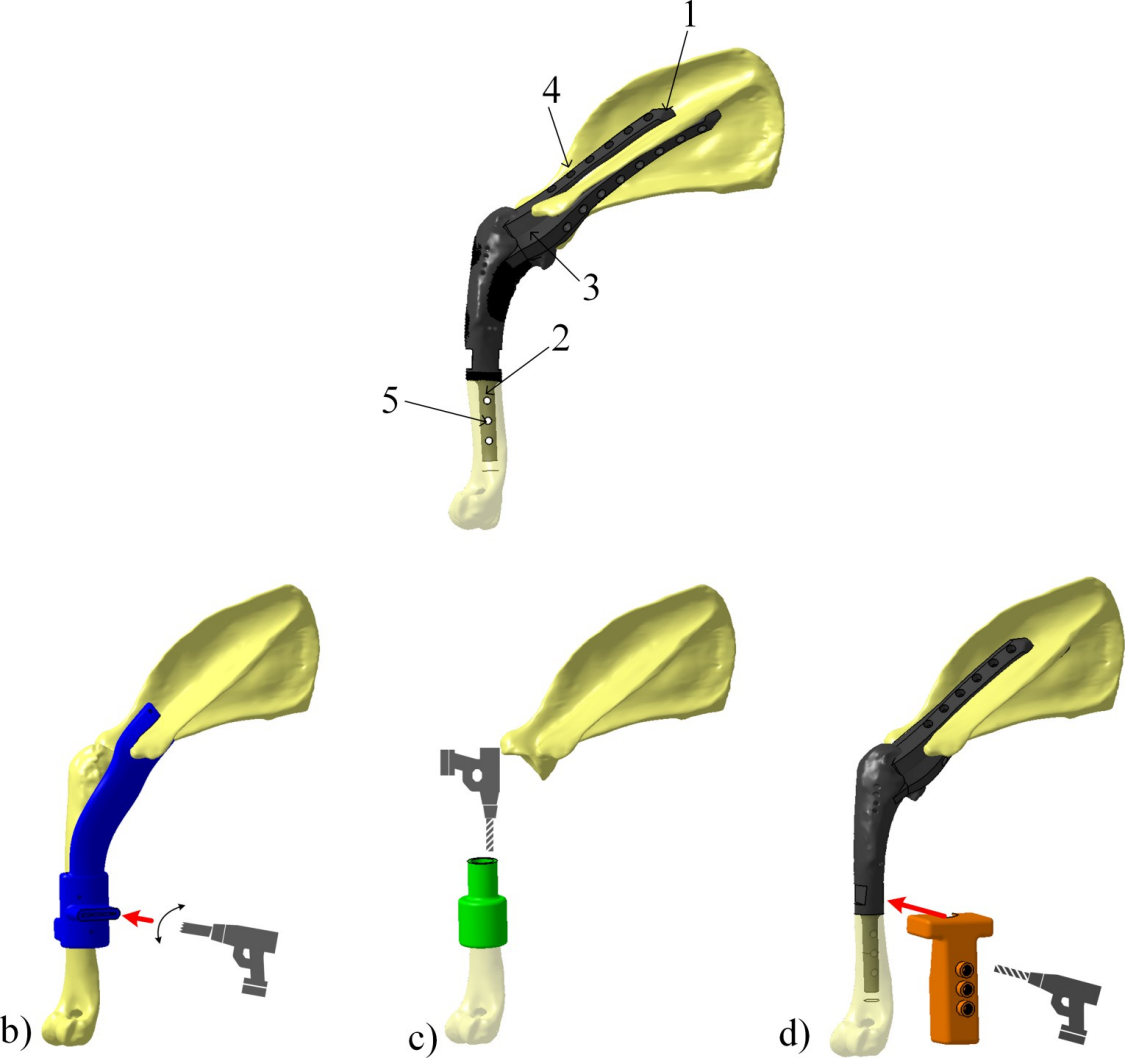
a) Personalized shoulder implant and surgical steps needed for its installation (excluding muscle reattachments): b) cutting of the excised part of the humerus, c) reaming of the intramedullary canal and d) drilling of the remaining part of the humerus [17].

This study proposes an approach to replace the affected segment of the proximal humerus with articulated or monobloc prostheses, with the objective of preserving the function of the salvaged limb. It is suggested to replace an excised portion of the proximal humerus affected by an osteosarcoma with a personalized prosthesis based on computed tomography of the limb [17]. The goal is to have the prosthesis span the bone defect caused by the surgical resection of the osteosarcoma, permit adequate biomechanical function of the spared limb, and finally minimize the risk of implant failure and infection. Such a prosthesis (Fig 1A) comprises three main functional components, namely, a scapular fixation (#1 in Fig 1), a humeral fixation (stem) (#2 in Fig 1) and a joint replica (#3 in Fig 1) extending between them. When in place, the scapular fixation is secured to the scapula using a series of self-tapered auto blocking cortical screws (#4 in Fig 1), the humeral stem is inserted in the medullary cavity of the humerus and also secured to the humerus with a series of self-tapered cortical screws (#5 in Fig 1). The design of the joint replica (#3 in Fig 1) determines the degree of the scapulohumerus relative movement. For example, the spherical joint is a ball-and-socket joint commonly used for shoulder and hip joint replacement. The socket surface covering the ball restricts the range of movement. The revolute joint contains a pin that can rotate along one axis (craniocaudal motion) with an abutment to limit the range of motion allowed by the prosthesis.

To ensure that the limb-sparing prosthesis fits the bone defect precisely in terms of length and position, cutting, reaming and drilling guides (namely CG, RG and DG) are used as shown in Fig 1B–1D. The cutting guide shape is influenced by the shape of the bone-tumor construct to be excised and the cutting guide length depends on the resection margin established by the surgeon to achieve a complete tumor resection. It also implies the passive components resection such as ligaments for instance. The drilling and reaming guides are shaped to conform to the part of the humerus that will remain after excision. At the end of the procedure, muscles must be attached to the prosthesis in specific zones (not illustrated therein) to ensure stability and different degrees of movement for the joint.

## Study methodology

**Fig 2:**
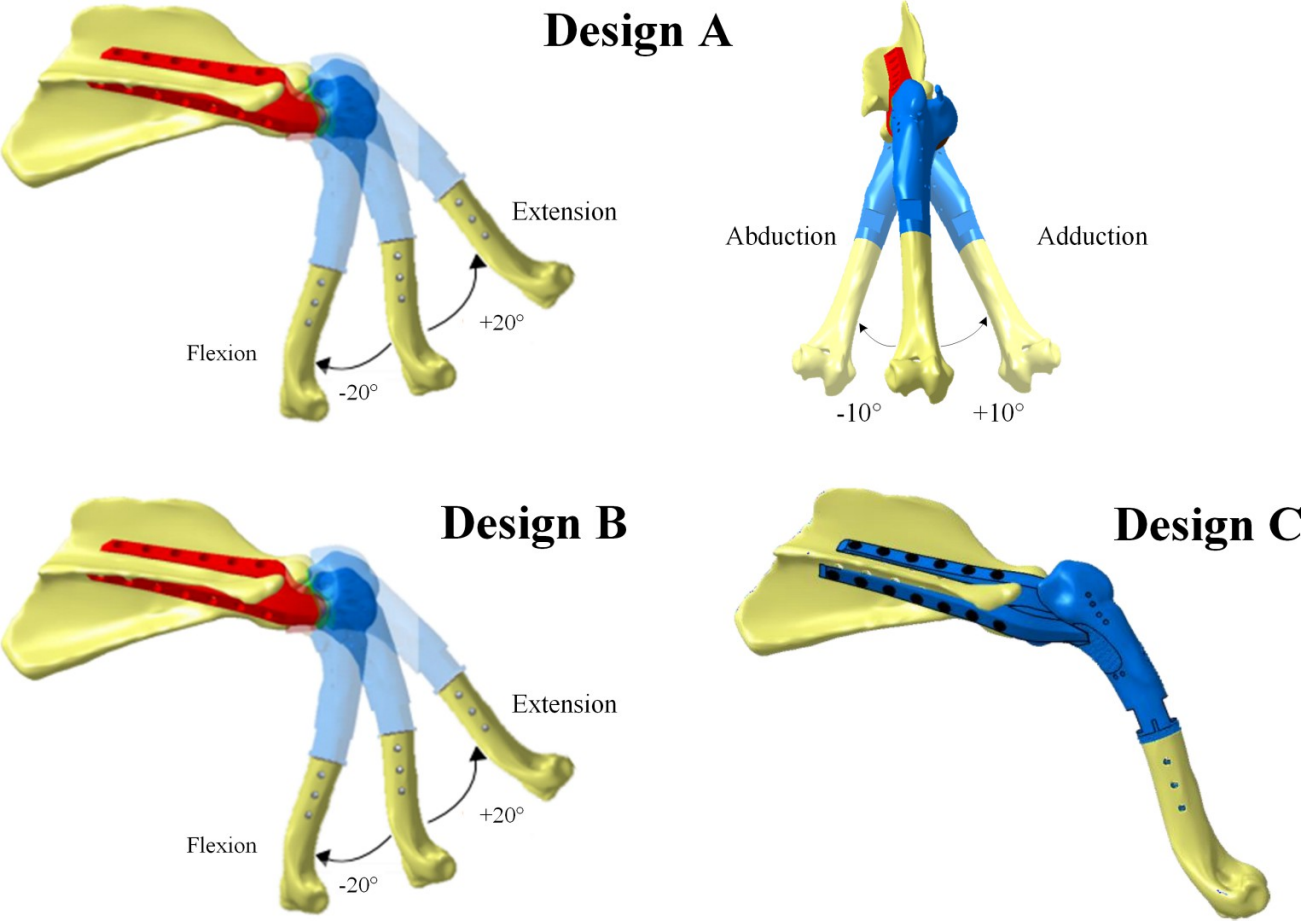
Design variants of the right thoracic limb prosthesis: Design A with spherical joint, Design B with revolute joint and Design C with monobloc prosthesis; degrees of mobility are taken from Nielsen, et al. [24].

Three different prosthesis designs providing varying degrees of mobility to the shoulder were developed. A prosthesis with a spherical connection (Fig 2 Design A) is intended to allow flexion/extension, adduction/abduction and internal/external rotation of the thoracic limb, and thus mimic the range of motion of the pre-existing shoulder joint and the joint stability, especially after the passive components resection. A prosthesis with a revolute joint allows a uniplanar limb flexion in the craniocaudal direction of the shoulder, but prevents adduction and abduction movements of the limb (Fig 2, Design B). Finally, a monobloc prosthesis that does not allow any movement between the scapula and humerus (Fig 2, Design C) is designed; it corresponds to the fusion of the shoulder joint (arthrodesis).

In order to ensure the functionality of each of the above presented prostheses and the corresponding biomechanical functions, the number of muscles to be attached to each of them, their contributions and roles within the scapulohumeral joint must be determined. These muscles and their functions in the healthy shoulder must be considered based on the state-of-the-art knowledge of shoulder biomechanics. In a healthy shoulder joint, the muscles must stabilize, flex, extend, adduct and abduct the thoracic limb, and can therefore be separated into three main categories according to their associated functions in the shoulder, namely, stabilizers, flexors/extensors and adductors/abductors [18]. The contributions of these muscles must be considered when the scapulohumeral joint, and therefore the movement between the scapula and humerus, are preserved (Design A and B, Fig 2) or sacrificed (Design C, Fig 2).

### Stabilizers

The *m*. *infraspinatus* and *m*. *biceps brachii* muscles are considered essential to stabilizing the dog’s shoulder joint [19]. The *m*. *infraspinatus* is mainly used as a stabilizer of the shoulder, while the *m*. *biceps brachii* helps stabilize the shoulder in a medial position in the extension position. *M*. *triceps lateralis* and *m*. *triceps medialis* also contribute to shoulder stability, but they mainly work against gravity [20].

### Flexors/Extensors

The locomotor functions of six muscles of the thoracic limb in flexion and extension positions (*m*. *pectoralis superficialis descendens*, *m*. *pectoralis profundus*, *m*. *latissimus dorsi*, *m*. *omostransversarius*, *m*. *cleidobrachialis* and *m*. *trapezius*) were evaluated by Carrier, et al. [21]. Among these six muscles, three are not attached to the humerus (*m*. *omostransversarius*, *m*. *cleidobrachialis* and *m*. *trapezius*), and are therefore not altered during the limb saving surgery. However, the three remaining muscles (*m*. *pectoralis superficialis descendens*, *m*. *pectoralis profundus and m*. *latissimus dorsi*) must be considered. The *m*. *latissimus dorsi* and *m*. *pectoralis profundus* muscles are involved in the flexion of the forelimb, while the *m*. *pectoralis superficialis* muscle allows adduction and extension of the front paw. Williams, et al. [22] identified the *m*. *latissimus dorsi* muscle as a generator of the shoulder flexion motion that plays a propulsive role. The *m*. *triceps brachii* serves to ensure the flexion of the shoulder and the stability of the joint, while the *m*. *biceps brachii* is involved in the extension of the shoulder in addition to its stabilizing function. In 2016, Araújo, et al. [23] evaluated the contributions of the thoracic limb muscles of boxer dogs and concluded that the *m*. *biceps brachii*, the *m*. *triceps brachii* and the *m*. *brachiocephalicus* all contribute to the walking of the dog, with the last one allowing the protraction (extension) of the shoulder [20].

**Fig 3:**
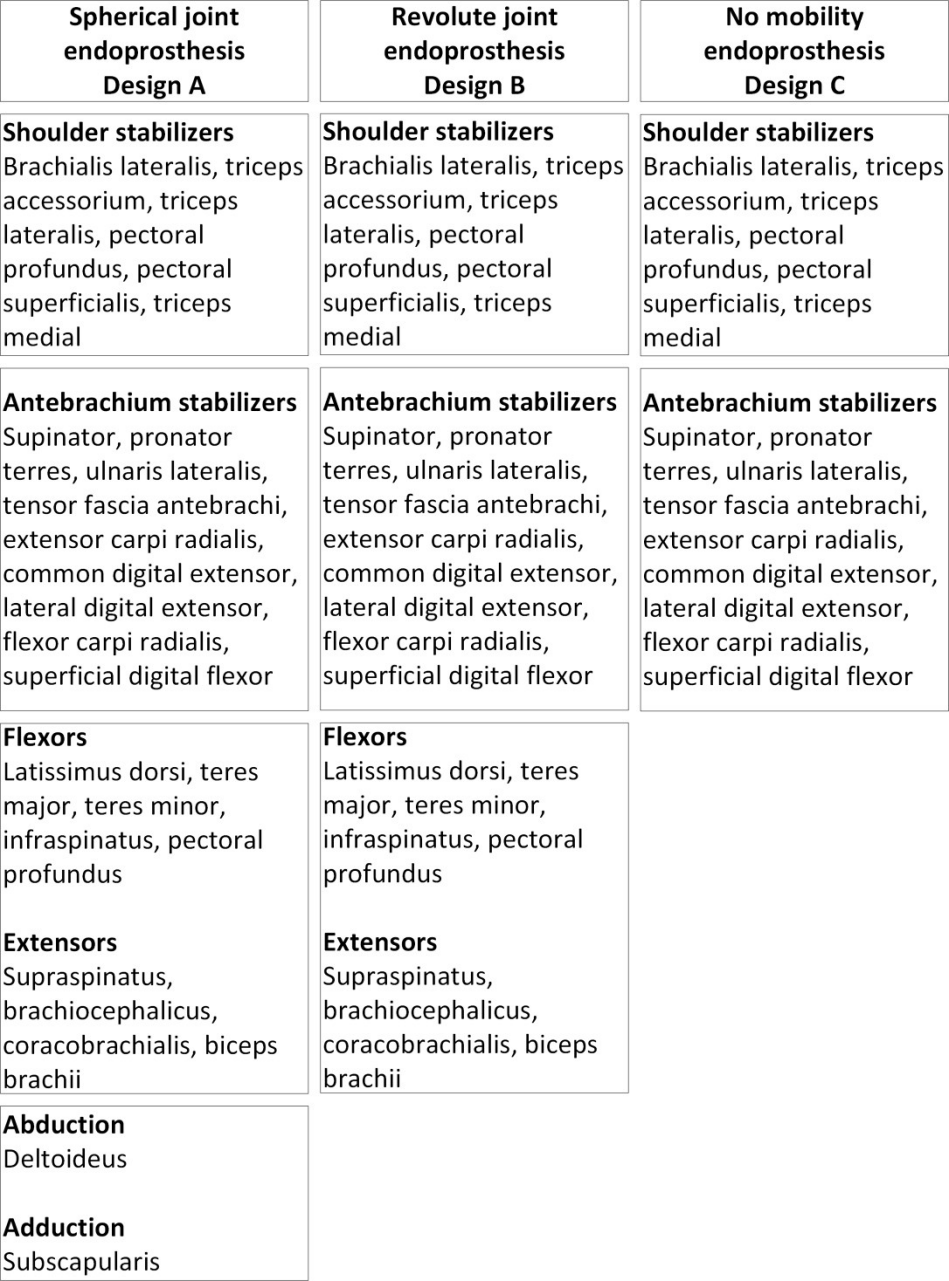
Synthesis scheme listing the muscles contributing to the prosthetic shoulder stability and mobility in the case of the different prosthesis designs: A, B and C.

Based on the above considerations, a subdivision of the muscles contributing to the stability and movements allowed by the different prosthesis designs is established and shown in Fig 3. Since different degrees of mobility are allowed by each of the prosthesis designs, the methodology of this study consists in building three simplified biomechanical models, one for each of the prosthesis designs by gradually removing degrees of mobility from Design A to Design B and to Design C. A minimum number of muscles required to preserve the shoulder functionality in each of the cases is also determined. For example, to switch from the prosthesis having a spherical connection A to that with a revolute connection B, the adduction and abduction movements are eliminated, and therefore, the muscles responsible for these movements no longer need to be preserved and attached to the prosthesis during the surgery. To determine the muscle strength contributions (in percentage of body weight, % BW), a quasi-static study of the joint biomechanics during walking is carried out. Note that the roles of the muscles located in the radius ulna site (elbow) are considered important for the thoracic limb stability, especially in the case of a monobloc prosthesis (prosthesis C, Fig 2C).

## Quasi-static study of the prosthetic shoulder biomechanics

**Fig 4:**
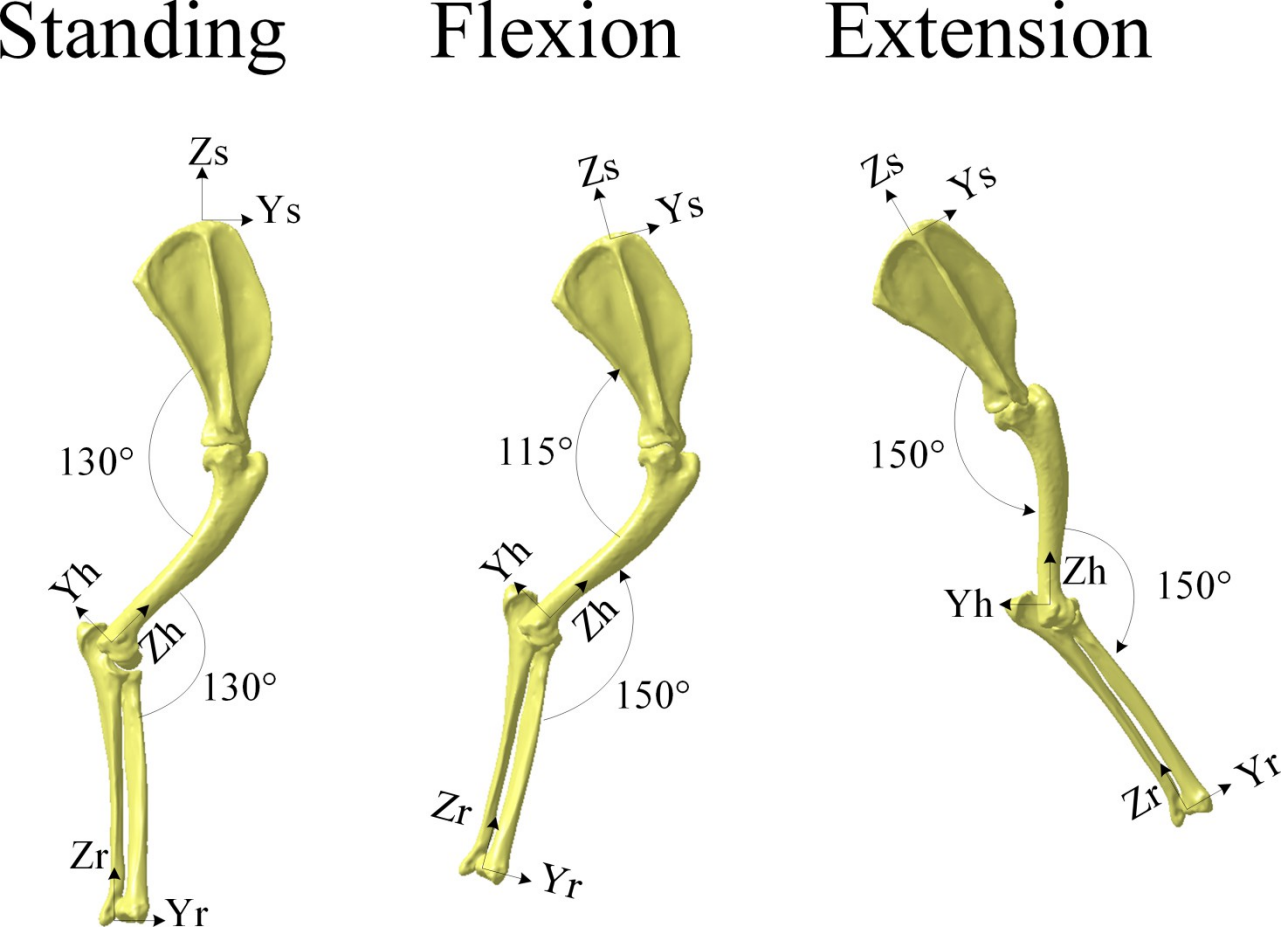
Location of the forelimb bones and their axis systems defined by Shahar and Milgram [14] in the thoracic limb.

This biomechanical study is limited to the assessment of the walking cycle of the prosthetic shoulder joint. To assess the recruitment of muscles of the front limb during walking, three distinct simulations are carried out by varying the range of motion of the shoulder joint. They are categorized as standing, flexion and extension positions of the front limb (Fig 4).

Based on the literature, the angle between the scapula and humerus is defined as follows: 130° for the standing position [23]; 115° for the flexion position and 150° for the extension position [24,25]. Furthermore, the angle between the humerus and radius/ulna is defined as follows: 130° for the standing position and 150° for both the flexion and extension positions [25] (Fig 4).

For all three positions (namely standing, flexion and extension), the same group of muscles is included in the simulation (Fig 3). As the dog’s walking speed can be considered low, the study can be reduced to a static analysis. Fixed landmarks in relation to the scapula, humerus and radius/ulna are defined in Fig 4. These coordinates allow to define the directions of forces associated with the muscles and their points of action on the bones.

The static equilibrium equations are then established, but since all the variables characterizing the muscle forces on the scapula and humerus are unknown, the musculoskeletal model presents different admissible solutions to the equilibrium constraints. Hence, minimizing the sum of the maximal muscle stresses could offer an appropriate solution of the optimization problem. Each solution represents a cost and the optimization criteria aims to find the minimum cost solution. The optimization criteria is based on the minimization of the maximal muscle stress (MMMS). This criteria is defined by minimization of *σ*, expressed as follows [26,27]:

$$\sigma =max_{i}\frac{\left|\vec{F_{i}}\right|}{PCSA_{i}}\left(\text{MMMS}\right)$$

where *i* = 1, 2, … for different muscles and PCSA is the physiological cross section area of each muscle determined according to:

$$PCSA=\frac{V.\text{cos}\left(\alpha \right)}{l}$$

where V represents the muscle volume, α the pennation angle and *l*, the muscle fiber length. The muscles’ PCSA values represent mean values adapted from Shahar and Milgram [14]. Data such as the muscles insertion and origin coordinates according to the bone landmarks were also extracted from that study (Table A in S1 Appendix). They were determined by cadaveric dissection of 23-kg mixed-breed male dogs. For the muscles not attached to the scapula, such as the *m*. *triceps accessorium*, *m*. *triceps lateralis*, *m*. *triceps medialis* and *m*. *brachialis lateralis*, coordinates in the humerus and radius/ulna reference system were used instead. In order to estimate their contributions in the shoulder environment, their coordinates were extrapolated by translations in the scapular landmark to include them in the simulations. The muscle contribution study was conducted using the MATLAB environment (2020a version), more specifically the optimal function from the optimization toolbox.

## Results

Static simulations were carried out for the prosthesis Designs A (spherical joint) and B (revolute joint) to identify the muscle contributions corresponding to the three positions of the stance phase: standing (130° angle between the scapula and the humerus), flexion (115° angle between the scapula and the humerus) and extension (150° angle between the scapula and the humerus). For the monobloc prosthesis Design C, only the standing position was considered, since implanting this prosthesis leads to shoulder arthrodesis and to a fixed angle between the scapula and the humerus.

### Spherical joint prosthesis (Design A)

**Fig 5:**
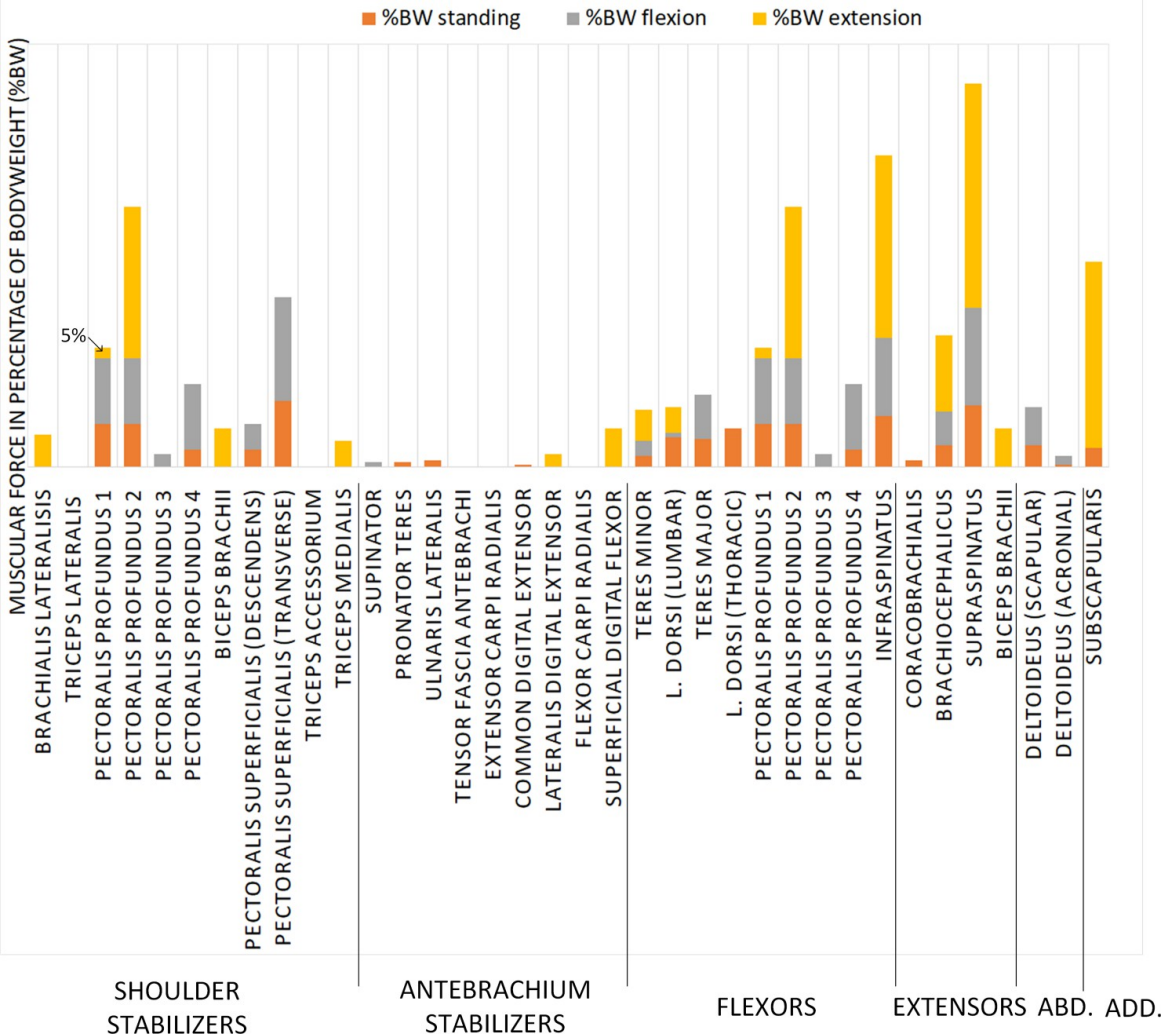
Force as a percentage of body weight, depending on the muscles recruited in different positions (flexion, extension, standing positions) for Design A. For each muscle, the muscular force is represented in the standing, flexion, and extension positions in absolute values. For each muscle, the muscle force values are not cumulative.

The muscles to be attached to the prosthesis with a spherical joint (Fig 3) are integrated into the simulation to assess their contribution in the standing, flexion and extension positions (Fig 4). Muscles with less than 5%BW of the force contribution were neglected and this cut-off value is based on the authors’ clinical expertise. In the standing position, among a total number of 31 muscles considered in the simulation, 13 appear as significant force generators, with a contribution exceeding 5%BW: *m*. *pectoral profundus 1*, *2* and *4*, *m*. *pectoralis superficialis descendens* and *transverse*, *m*. *latissimus dorsi lumbar* and *thoracis*, *m*. *teres major*, *m*. *infraspinatus*, *m*. *brachiocephalicus*, *m*. *supraspinatus*, *m*. *deltoideus scapular* and *m*. *subscapularis* (Table 1). It is found that 41% of the recruited muscles are stabilizer muscles in this position. In the flexion position, 12 muscles are recruited, with 6 of them being flexor muscles. Their contribution represents 54% of the total generated muscular force for this position. In the extension position, 13 muscles are recruited, with 3 of them being the extensor muscles. Their contribution represents 32% of the total generated muscular force. Abductor muscles are all solicited in the standing and flexion positions, but not in the extension position, and the adductor muscles are recruited in the standing and extension positions (Fig 5). From the simulation results, muscles that do not contribute as force generators for each position are the following ones: *m*. *triceps lateralis*, *m*. *triceps accessorium*, *m*. *tensor fascia antebrachii*, *m*. *extensor carpi radialis* and *m*. *flexor carpi radialis*. As the simulation converged with those results, the stability criterion has been reached with this force distribution. Consequently, it would theoretically not be necessary to reattach them to this prosthesis design.

**Table 1:** Simulation results for the muscular environment of the prosthesis with spherical joint, Design A. The intensity of the grey color indicates the importance of the muscle force on the articulation. Inactive muscles are indicated by a dash; %BW signifies muscle contributions in the percentage of body weight.

Groups	Muscles	%BW standing	%BW flexion	%BW extension
Shoulder stabilizers	*brachialis lateralis*	-	-	15
	*triceps lateralis*	-	-	-
	*pectoralis profundus 1*	20	31	5
	*pectoralis profundus 2*	20	31	72
	*pectoralis profundus 3*	-	6	-
	*pectoralis profundus 4*	8	31	-
	*biceps brachii*	-	-	18
	*pectoralis superficialis (descendens)*	8	12	-
	*pectoralis superficialis (transverse)*	31	49	-
	*triceps accessorium*	-	-	-
	*triceps medialis*	-	-	12
Antebrachium stabilizers	*supinator*	-	2	-
	*pronator teres*	2	-	-
	*ulnaris lateralis*	3	-	-
	*tensor fascia antebrachi*	-	-	-
	*extensor carpi radialis*	-	-	-
	*common digital extensor*	1	-	-
	*lateralis digital extensor*	-	-	6
	*flexor carpi radialis*	-	-	-
	*superficial digital flexor*	-	-	18
Flexors	*teres minor*	5	7	15
	*latissimus dorsi (lumbar)*	14	2	12
	*teres major*	13	21	-
	*latissimus dorsi (thoracis)*	18	-	-
	*pectoralis profundus 1*	20	31	5
	*pectoralis profundus 2*	20	31	72
	*pectoralis profundus 3*	-	6	-
	*pectoralis profundus 4*	8	31	-
	*infraspinatus*	24	37	86
Extensors	*coracobrachialis*	3	-	-
	*brachiocephalicus*	10	16	36
	*supraspinatus*	29	46	106
	*biceps brachii*	-	-	18
Abductors	*deltoideus (scapular)*	10	18	-
	*deltoideus (acromial)*	1	4	-
Adductors	*subscapularis*	9	-	88

### Revolute joint prosthesis (Design B)

The prosthesis with a revolute joint does not allow adduction nor abduction movements, and therefore, the muscles responsible for these movements are removed from the simulation. Of the 28 muscles included in the simulation, 11 exert a force on the shoulder joint when the dog is standing when considering a contribution exceeding 5%BW: *m*. *pectoral profundus 1*, *2 and 4*, *m*. *pectoralis superficialis descendens and transverse*, *m*. *latissimus dorsi lumbar and thoracis*, *m*. *teres major*, *m*. *infraspinatus*, *m*. *brachiocephalicus and m*. *supraspinatus* (Table 2).

**Table 2:** Simulation results for the muscular environment of the prosthesis with a revolute joint, Design B. The intensity of the grey color indicates the importance of the muscle force on the articulation. Inactive muscles are indicated by a dash.

Groups	Muscles	%BW standing	%BW flexion	%BW extension
Shoulder stabilizers	*brachialis lateralis*	-	-	-
	*triceps lateralis*	5	18	-
	*pectoralis profundus 1*	20	35	-
	*pectoralis profundus 2*	20	35	-
	*pectoralis profundus 3*	-	-	-
	*pectoralis profundus 4*	11	31	-
	*biceps brachii*	-	1	33
	*pectoralis superficialis (descendens)*	8	14	-
	*pectoralis superficialis (transverse)*	31	39	-
	*triceps accessorium*	-	-	-
	*triceps medialis*	-	-	-
Antebrachium stabilizers	*supinator*	2	-	-
	*pronator teres*	2	4	-
	*ulnaris lateralis*	3	-	6
	*tensor fascia antebrachi*	-	-	-
	*extensor carpi radialis*	5	8	-
	*common digital extensor*	5	-	-
	*lateralis digital extensor*	-	-	11
	*flexor carpi radialis*	-	-	-
	*superficial digital flexor*	-	1	33
Flexors	*teres minor*	5	8	-
	*latissimus dorsi (lumbar)*	14	-	-
	*teres major*	13	24	-
	*latissimus dorsi (thoracis)*	18	-	-
	*pectoralis profundus 1*	20	35	-
	*pectoralis profundus 2*	20	35	-
	*pectoralis profundus 3*	-	-	-
	*pectoralis profundus 4*	11	31	-
	*infraspinatus*	24	42	160
	*coracobrachialis*	2	-	11
Extensors	*brachiocephalicus*	10	18	51
	*supraspinatus*	29	52	25
	*biceps brachii*	-	1	33

**Fig 6:**
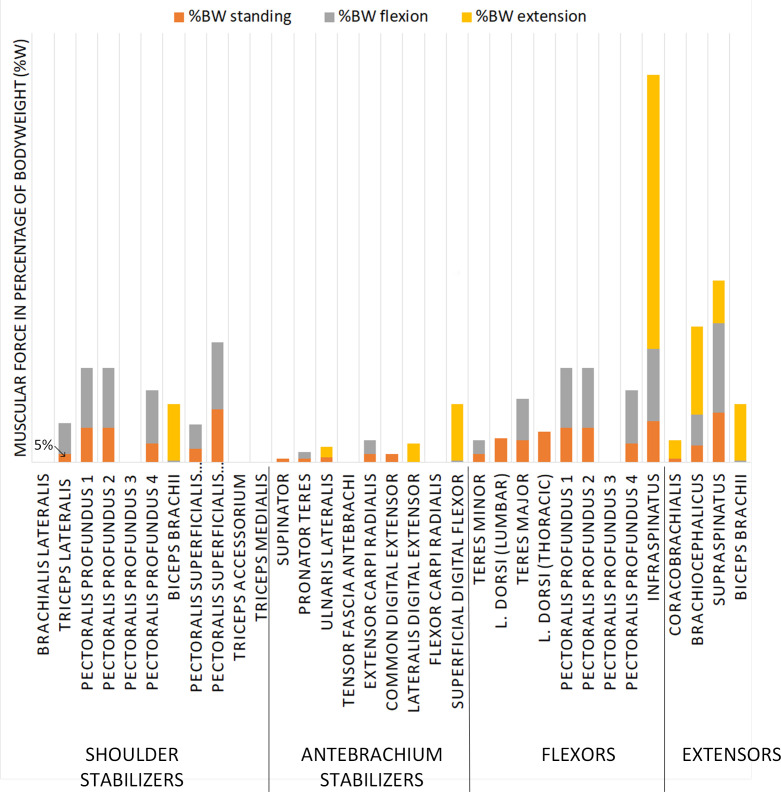
Force as a percentage of body weight depending on the muscles recruited in different positions (flexion, extension, standing positions) for Design B. For each muscle, the muscular force is represented in the standing, flexion and extension position in absolute values. For each muscle, the muscle force values are not cumulative.

The standing and flexion positions recruit more muscles than does the extension position, and most of the recruited muscles in the extension position are naturally extensors. About 45% of the total muscle force is generated by stabilizer muscles in the standing and flexion positions, and 41% of the total muscle force is activated by flexor muscles in the flexion position. Even though only 33% of the total generated muscular force is recruited by extensor muscles in the extension position, of the 9 muscles recruited in this position, 5 are extensors. Moreover, *m*. *infraspinatus* identified as a flexor muscle, shows a predominant force contribution in flexion (Fig 6). Its relative contribution is therefore more important than in the spherical joint prosthesis. Since abductor and adductor muscles are removed, the force distribution between the muscles changes. From these results, muscles that do not contribute as force generators for each position are the following ones: *m*. *brachialis lateralis*, *m*. *pectoralis profundus 3*, *m*. *triceps accessorium*, *m*. *triceps medialis*, *m*. *tensor fascia antebrachi* and *m*. *flexor carpi radialis*. With the stability criterion reached, it would not be necessary to reattach them to this prosthesis design.

### Monobloc (no shoulder mobility) prosthesis (Design C)

**Fig 7:**
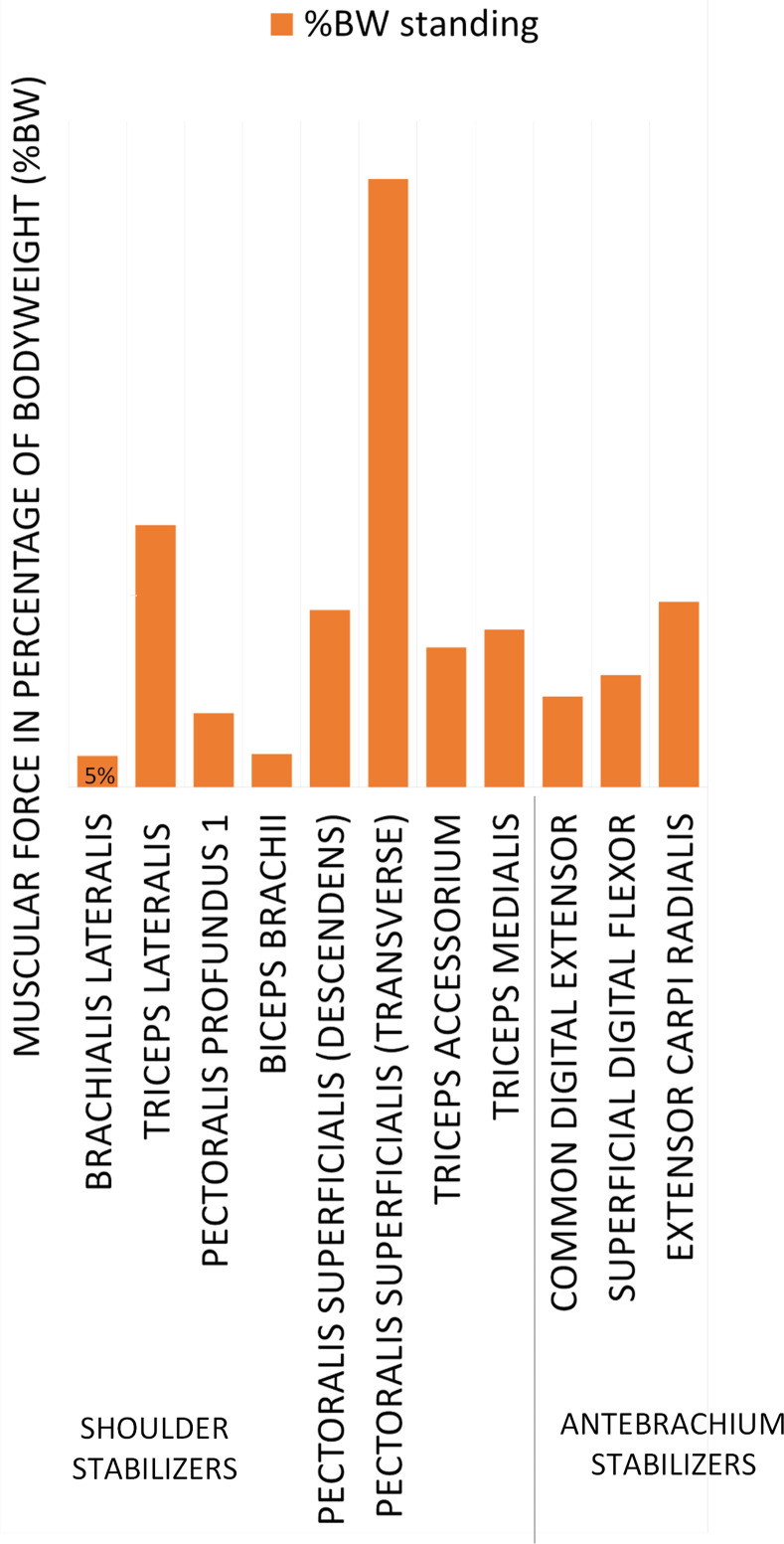
Strength as a percentage of body weight depending on the muscles recruited for Design C. For each muscle, the muscular force is represented in the standing position in absolute values.

For this design configuration, the shoulder joint is considered fixed since the prosthesis is a monobloc. The muscles attached only between the humerus and the scapula are therefore removed from the simulations, since mobility is lost between these two bones. The forces exerted by the muscles on the radius, ulna and humerus are calculated. Only the elbow joint generates a movement of the front limb in the case of the shoulder arthrodesis, caused by the prosthesis design. The simulation results shown in Table 3 seem to confirm the assumptions made in Fig 3. Since all the selected muscles exert a force on the humerus (Fig 7), it can be assessed that they should all be attached to the prosthesis.

**Table 3:** Simulation results for the muscular environment of the prosthesis with no mobility allowed, Design C.

Groups	Muscles	%BW standing
Shoulder stabilizers	*brachialis lateralis*	5
	*triceps lateralis*	39
	*pectoralis profundus 1*	11
	*biceps brachii*	5
	*pectoralis superficialis (descendens)*	27
	*pectoralis superficialis (transverse)*	91
	*triceps accessorium*	21
	*triceps medialis*	24
	*common digital extensor*	14
Antebrachium stabilizers	*superficial digital flexor*	17
	*extensor carpi radialis*	28

### Muscles fixation

**Fig 8:**
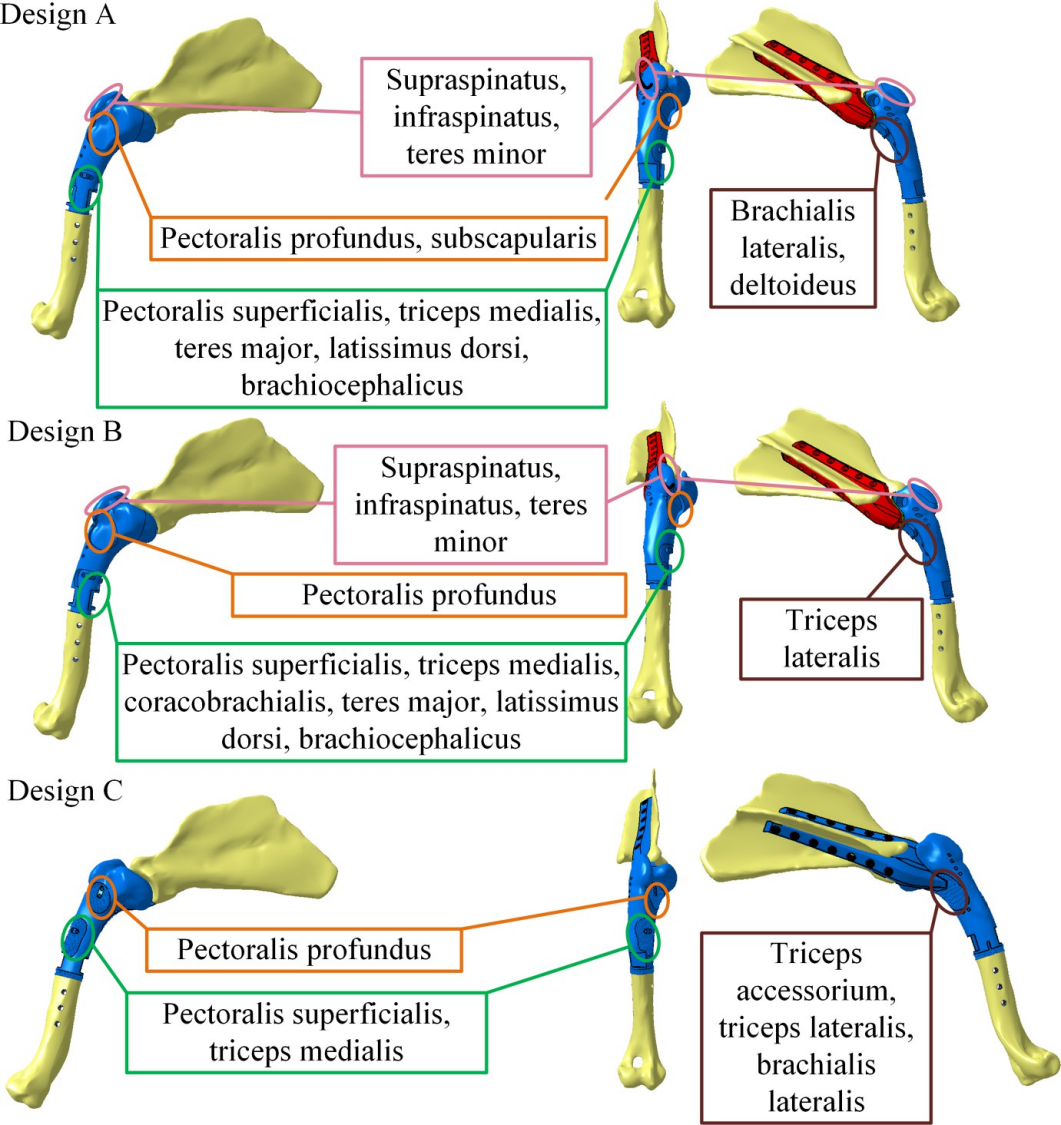
Identified areas of the muscle reattachments to the prostheses after the limb saving surgery: Design A (spherical joint), Design B (revolute joint) and Design C (no mobility).

The results obtained using a simplified model of the shoulder biomechanics help identify the muscles to be prioritized for reattachment to the prostheses A, B and C after the limb-saving surgery. To preserve the shoulder functionality as much as possible, sites where the muscles are attached to the prostheses must remain similar to their positions in the physiologic joint [18]. Additional simulations were carried out with a ±10% deviation between the muscle origin and insertion coordinates to assess the %BW variability. No significant changes in the distribution of the force contributions were observed. Consequently, even if the muscle fixation procedure does not exactly respect the initial attachment points of muscles, the muscle force contributions found by the numerical simulations should remain the same. Since the degree of mobility offered by each of the prosthesis designs differ, the number of muscles needing to be reattached will also be different and grouped together as shown in Fig 8. Results from Table 1 confirm that antebrachium stabilizers muscles do not need to be attached to the design A as they do not contribute significantly to force generation, considering the 5%BW cut-off value. When comparing Designs A and B, only the *m*. *subscapularis* and *m*. *deltoideus* are removed. It was observed from Figs 5 and 6 that there were no rearrangements in the muscles’ activation and that the loss of the abductor and adductor muscles (*m*. *deltoideus*, *m*. *subscapularis*) is compensated by a more significant recruitment of the antebrachium stabilizer muscles. Comparing Designs A and B, we see that the same muscle fixation zones are required but fewer muscles need to be reattached in the case of Design B. For Design C, the adductor, abductor, flexor and extensor muscles are removed from the muscle fixation zones. However, more stabilizer muscles need to be reattached to the muscle fixation zones common to Designs A and B, in order to ensure limb stability.

## Discussion

The objective of this study was to use the quasi-static biomechanical analysis as a decision-making tool for the limb-sparing surgery in the case of osteosarcoma of the proximal humerus in dogs. To this end, the muscles contributions within the shoulder joint were assessed and the ones needing to be reattached to a scapulohumeral joint prosthesis were identified, thus ensuring either total or partial joint mobility, or only joint stability, depending on the specific design of the prosthesis. The biomechanical analysis led to the following findings and interpretations.

### Spherical joint prosthesis (Design A)

The assumption regarding the muscles to be attached to the prosthesis with a spherical connection is confirmed: the flexion and extension of the limb recruit the muscles responsible for these movements. The physiologic shoulder model allowing flexion/extension and adduction/abduction movements uses coherent muscles for different angular positions between the scapula and the humerus. As expected, most of the flexor muscles are recruited in the flexion position, while most of the extensors are recruited in the extension positions. It appears that *m*. *supraspinatus*, which is a predominantly extensor muscle, contributes mainly in the extension position, while its contribution is significantly reduced in the flexion and standing positions. This muscle is recruited to reach the extension position and becomes the most stressed muscle in that position. It was found previously that *m*. *infraspinatus* and *m*. *supraspinatus* show the largest activation during the walking gait and are activated during the whole stance phase [20]. This is also the case of this study. It appears that the *m*. *triceps accessorium* and the *m*. *triceps lateral* do not generate any force. It was also ascertained from the study by Stark, et al. [20] that the *m*. *triceps accessorium* and the *m*. *triceps lateralis* do not generate any force, and so it would not be necessary to reattach these muscles to this type of prosthesis. However, from the clinical expertise point of view, *m*. *triceps lateralis* muscle is necessary to maintain stance as it contributes to elbow extension, that is why it was chosen to insert in to the design A (Fig 8). With their insertion in the radius/ulna region, the muscles that are supposed to stabilize the antebrachium, are not significantly solicited with this type of prosthesis. Muscles with a force contribution less than 5% are mainly part of this group: *m*. *supinator*, *m*. *pronator teres*, *m*. *ulnaris lateralis*, *m*. *common digital extensor*, *m*. *coracobrachialis* and *m*. *deltoideus acromial*.

### Revolute joint prosthesis (Design B)

It is observed that all 28 muscles considered in the simulations are involved in the prosthetic shoulder function, but their relative contributions depend on the position of the limb. The stabilizing muscles are more stressed in the revolute joint prosthesis, Design B (Fig 6), than in the spherical joint prosthesis, Design A (Fig 5). Similarly to Design A, the *m*. *triceps accessorium* muscle does not generate any force in Design B. However, compared to Design A, more shoulder and antebrachium stabilizers muscles are recruited to compensate for the loss of the abductor/adductor muscles. Their greater contribution helps to stabilize the thoracic limb. In the extension position of the thoracic limb, the *m*. *teres major* muscle (flexor muscle) is no longer solicited, while the *m*. *biceps brachii* muscle become more solicited as compared to the standing position. The function of the *m*. *biceps brachii* is to extend the shoulder and stabilize the shoulder [19]. The stabilizing muscles of the shoulder are even more necessary as the *m*. *deltoid* and *m*. *subscapularis* muscles are no longer considered within the joint.

### Monobloc (no shoulder mobility) prosthesis (Design C)

When considering only the muscles of the shoulder, no solution was found to meet the equilibrium constraints stemming from the Static Fundamental Principle. It was found that by adding three other muscles (*m*. *common digital extensor*, *m*. *superficial digital flexor*, *m*. *extensor carpi radialis*) located in the radius ulna/humerus joint, the static equilibrium could be respected. These muscles were selected due to the belief that muscles from the elbow joint could help stabilize the joint and be reattached to the prosthesis’ humerus distal part. Besides, the simulation showed that these muscles are significant force generators. While the *m*. *triceps accessorium* was not considered as a force generator for Designs A and B, its recruitment is observed in Design C, when the shoulder joint is sacrificed, in order to compensate for the loss of muscles responsible for the flexion and extension movements.

### Study limitations

This study has certain limitations, with the main one being considering muscles acting along straight lines between their insertion points on bones. Also, muscles to be salvaged during the surgery were defined to ensure thoracic member stability during locomotion and not idiomotion. The main goal of personalized endoprotheses presented in this study consists in improving the quality of life, by providing a reasonable mobility to the affected healing thoracic limb. Progressive function restoration is prioritized instead of targeting to maximize the range of motion and movement amplitude. That is why other natural’s dog movement (digging, scratching) require a special study and are not included here. This study is based on cadaveric measurements data whose accuracy is a function of the dissection method used. Consequently, the next study should use MRI scans in order to leverage morphological data from medical imaging instead of using literature data [14]. Doing so, it is expected to compare cadaveric, imaging and numerical data for a given specimen.

## Conclusions

Although osteosarcoma commonly affects the proximal humerus, the optimal limb-sparing surgery technique has not been yet reported, and amputation is the solution chosen by default. Additive manufacturing is a fabrication process that enables to offer patient-specific prostheses, thus ensuring that the medical device could be perfectly adapted to the patient’s morphology. In this study, it is proposed to replace the affected humerus with a patient-specific humerus/scapula joint prosthesis, the latter having three alternative designs: a) a spherical joint prosthesis, b) a revolute joint prosthesis, and c) a monobloc prosthesis. Moreover, to ensure the prosthetic joint functionality, some muscles need to be reattached to it during surgery. To determine the muscles to be salvaged, we carried out a biomechanical study determining the contributions of the shoulder muscles for different prosthesis designs and phases of the canine gait cycle. Results showed that all the muscles integrated in the simulation involving prosthesis with spherical joint design are activated in the standing (130°), flexion (115°) and extension (150°) positions. Muscular forces mainly increase as we go from the standing position to the flexion position, in which the most solicited muscles are indeed the flexors. From the standing to the extension positions, there is a rearrangement of the muscular forces. The *m*. *supraspinatus* muscle is recruited and becomes the most solicited muscle in the extension position. In this position, the number of activated extensor muscles increases, while the number of activated flexor muscles decreases. Every muscle is solicited depending on its specific position between the bones, and their attachment to the prosthesis is then required to ensure every movement of the shoulder articulation. This study illustrates that the *m*. *triceps accessorium* muscle does not need to be reattached to Design A and Design B prostheses. In the simulations involving the prosthesis with a revolute joint design, a more homogeneous distribution of the muscular forces is observed, compensating for the loss of the adductor and abductor muscles. According to the muscles’s locations and contributions, their reattachment areas were identified. Nevertheless, further investigations are needed in order to assess the behavior of the muscles reattached on prostheses with the help of structured porous-like fixation zones. It is not yet certain whether such printed lattice structures would ensure full mobility of the canine thoracic limb.

## Supporting information

S1 Fig (TIF)

S1 File (DOCX)

S1 Appendix (DOCX)
