# Supplementary figures and images for: Personalized endoprostheses for the proximal humerus and scapulohumeral joint in dogs: Biomechanical study of the muscles’ contributions during locomotion
Source: PLoS One. 2022 Jan 24;17(1):e0262863. doi: 10.1371/journal.pone.0262863 (PMC8786195; doi:10.1371/journal.pone.0262863)

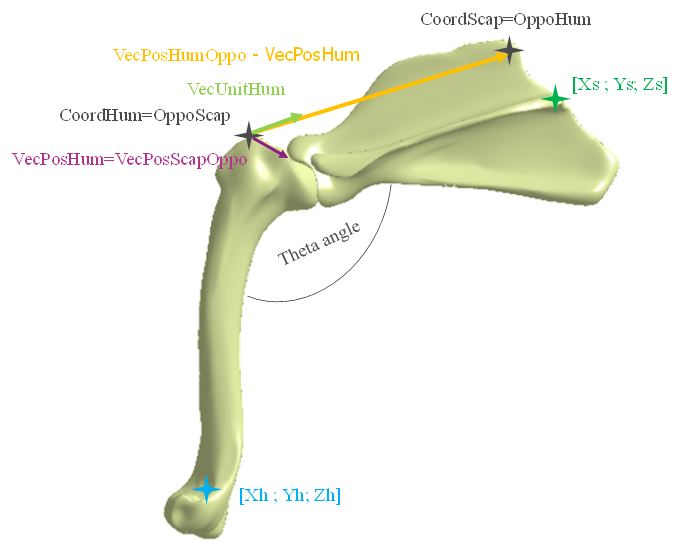

Supplement: S1 Fig — (TIF) [file pone.0262863.s001.tif]
