# Supplementary material for: Personalized endoprostheses for the proximal humerus and scapulohumeral joint in dogs: Biomechanical study of the muscles’ contributions during locomotion
Source: PLoS One. 2022 Jan 24;17(1):e0262863. doi: 10.1371/journal.pone.0262863 (PMC8786195; doi:10.1371/journal.pone.0262863)
Supplement: S1 File — (DOCX) [file pone.0262863.s002.docx]

**Supporting information**

The computation method is based on morphometric data shared by [[28](#_ENREF_28)] and allows to estimate each muscle force (in percentage of body weight) involved in the canine shoulder joint. The following line codes were used for the design configuration A and B muscles force estimation. The code script requires Matlab to be computed and an excel files with morphological data (table A in the appendix). Some functions such as *VectPos*, *CorrectOppo* and *Normalize* are given from L. 641 to L.693 as scripts especially created for the code.

The first step consists in defining the relative position bones of the thoracic limb, their corresponding landmarks and the rotation center coordinates, expressed in the scapula and humerus respective landmarks.

% rotation center coordinates

C_rot = [ Xh Yh Zh ; Xs Ys Zs] ;

% theta angle between Zs and Zx axis

theta = ϴ/180*pi ;

Then, muscles data are incorporated from an excel files with PCSA, insertion and origin coordinates, that corresponds to table A.

% [num, txt, tab] = xlsread('fichier.xls');

numHum = xlsread('humerus.xlsx') ;

numScap = xlsread('scapula.xlsx') ;

coordHum = numHum(:,1:3); %coord of the insertion point on the bone

oppoHum = numHum(:,4:6); %coord of the opposite insertion point

PCSAHum = numHum(:,end); %value of the muscle's PCSA

nb_Hum = size(numHum,1); %number of muscles on the humerus

coordScap = numScap(:,1:3);

oppoScap = numScap(:,4:6);

PCSAScap = numScap(:,end);

nb_Scap = size(numScap,1);

A list is then generated to obtain for the muscle *i* that is inserted to the point *P_i,_* the position vector *VecPos* according to the rotation center $\vec{CrotPi}$, in the according landmark (scapula and humerus).

VecPosHum = VectPos(coordHum, C_rot,'humerus');

VecPosScap = VectPos(coordScap, C_rot,'scapula');

Unit vectors that indicate the line of action (direction force) of the muscles are computed.

VecPosHumOppo = VectPos(oppoHum, C_rot,'scapula');

VecPosHumOppo = correctOppo(VecPosHumOppo, theta, 'humerus'); %compute correction

VecPosScapOppo = VectPos(oppoScap, C_rot,'scapula');

VecPosScapOppo = correctOppo(VecPosScapOppo, theta, 'scapula'); %compute correction

VecUnitHum = normalize(VecPosHumOppo - VecPosHum) ;

VecUnitScap = normalize(VecPosScapOppo - VecPosScap) ;

The *correctOppo* function allows to redirect vectors in the correct base.

**S1 Fig. Supporting information**

A vector for the ground reaction is calculated in order to calculate the body weight over the thoracic limb. This reaction is assumed to be vertical: an 45° angle is formed between humerus axis and the axis that is perpendicular to the ground.

verticalUnit = [0 -sqrt(0.5) sqrt(0.5)];

BW_pourcentage = 65

BWPos = -C_rot(2,:) ;

Then, the equation system must be introduced. When considering two rigid bodies (scapula and humerus) with negligible mass, the study is limited to a quasi-static case. Force modulus exerted by the muscles represent the unknowns variables of the static problem.

%x=optimvar('x',dim1,dim2,dim3,'Type','integer','LowerBound',minValue,'UpperBound',maxValue)

F_scapula = optimvar('F_scapula',nb_Scap,1,'LowerBound',0);%muscles modulus

F_hum_scap = optimvar('F_hum_scap',3,1); % reaction vector components

F_humerus = optimvar('F_humerus',nb_Hum,1,'LowerBound',0); %muscles modulus

F_scap_hum = optimvar('F_scap_hum',3,1); % reaction vector components

Forces direction of each muscle is known, as for their action point on bones. Consequently, only muscles force modulus and reaction forces between scapula and humerus are to be calculated.

Static fundamental principle :

$\sum_{i \in scapula} \vec{F_{i}}=0$  (1)

$\sum_{i \in scapula} \vec{F_{i}}\bigwedge\vec{r_{i}}=0$   (2)

$\sum_{i \in humerus} \vec{F_{i}}=0$   (3)

$\sum_{i \in humerus} \vec{F_{i}}\bigwedge\vec{r_{i}}=0$  (4)

% Static fundamental principle equations

eqn1 = VecUnitScap' * F_scapula + F_hum_scap == 0;

eqn2 = (cross(VecUnitScap,VecPosScap))' * F_scapula == 0;

eqn3 = VecUnitHum' * F_humerus + BW_pourcentage*verticalUnit' + F_scap_hum == 0;

eqn4 = (cross(VecUnitHum,VecPosHum))' * F_humerus + BW_pourcentage*(cross(verticalUnit,BWPos))' == 0;

Considering the opposition between muscular efforts, the following equation are computed:

% Modulus equivalence

eqn5 = F_humerus( :) == F_scapula(:) ;

$\vec{F_{hum scap}}= -\vec{F_{scap hum}}$    (5)

% Rotation matrix from humerus to scapula landmark

R = [1 0 0 ;

0 cos(theta) sin(theta);

0 -sin(theta) cos(theta)] ;

eqn6 = F_hum_scap == - R*F_scap_hum ; (6)

The quasi-static problem is solved by optimization criteria.

With the help of Shahar data, unit vectors define direction of muscles action and position vectors that link the rotation center to the insertion point of each muscle to the bone could be defined. After establishing every equation and equivalence relationships between forces modulus, it is found that there are more unknowns variables for the number of equations. There is then an infinite number of solution. In order to solve this problem, optimization criteria is used in order to respect the problem constraints (from equation 1 to 8).

z = optimvar('z',1,1);

A = F_humerus./PCSAHum ;

B = F_scapula./PCSAScap ;

eqn7 = z >= A ; (7)

eqn8 = z >= B ; (8)

% definition of the MMMS problem

prob2=optimproblem;

prob2.Objective = z ; %optimization MMMS

prob2.Constraints.cons1 = eqn1;

prob2.Constraints.cons2 = eqn2;

prob2.Constraints.cons3 = eqn3;

prob2.Constraints.cons4 = eqn4;

prob2.Constraints.cons5 = eqn5;

prob2.Constraints.cons6 = eqn6;

prob2.Constraints.cons7 = eqn7;

prob2.Constraints.cons8 = eqn8;

%VectPos===================================================================

function [Vpos] = VectPos(coord,C_rot, s_h)

% coord is a matrix with the coordinates of the insertion % points of the muscles.

% C_rot is a 2*3 matrix with the coordinates of the rotation % center.

% s_h is a string 'scapula' or 'humerus'

c = 1 ;

Vpos = zeros(size(coord,1),3);

if s_h == 'scapula'

rot = C_rot(1,:);

elseif s_h == 'humerus'

rot = C_rot(2,:);

else

c = 0;

disp('s_h must be a string (scapula, or humerus)');

end

if c == 1

Vpos = coord - rot;

end

end

%CorrectOppo===============================================================

function [VecPosOppo] = correctOppo(VecPosOppo, theta, s_h)

% give the coordinates of the opposite insertion points in the correct system

% C_rot is a 2*3 matrix with the coordonates of the rotation % center.

% s_h is a string 'scapula' or 'humerus'

c = 1 ;

Rotation = [ 1 0 0 ;

0 cos(theta) -sin(theta);

0 sin(theta) cos(theta)] ;

if s_h == 'scapula'

% rotation of humerus landmark to scapula landmark

R = Rotation' ;

elseif s_h == 'humerus'

% rotation of scapula landmark to humerus landmark

R = Rotation ;

else

c = 0;

disp('s_h must be a string (scapula, or humerus)');

end

VecPosOppo = VecPosOppo*(R') ;

End

%Normalize================================================================

function [unit_vector] = normalize(Vector)

% normalize the vector

norm = sqrt(sum(Vector.^2,2));

unit_vector = Vector./norm;

end
