# Supplementary material for: Personalized endoprostheses for the proximal humerus and scapulohumeral joint in dogs: Biomechanical study of the muscles’ contributions during locomotion
Source: PLoS One. 2022 Jan 24;17(1):e0262863. doi: 10.1371/journal.pone.0262863 (PMC8786195; doi:10.1371/journal.pone.0262863)
Supplement: S1 Appendix — (DOCX) [file pone.0262863.s003.docx]

# **Appendix**

Table A: Coordinates of the muscles’ origins, insertion points and PCSAs in the shoulder environment of a dog adapted from [Shahar and Milgram [14]](#_ENREF_14).

| Groups | Muscle | Xs | Ys | Zs | Xh | Yh | Zh | Xru | Yru | Zru | PCSA |
| --- | --- | --- | --- | --- | --- | --- | --- | --- | --- | --- | --- |
| Shoulder stabilizers | brachialis lateralis | ***7,03*** | ***374*** | ***372,94*** | -2,01 | -11,52 | 151,11 | -7,03 | -1,06 | 0 | 1,04 |
|  | triceps accessorium | ***-9,91*** | ***374*** | ***357,15*** | -18,79 | -18,59 | 145,87 | 9,91 | -16,85 | 0 | 1,85 |
|  | triceps lateralis | ***-9,91*** | ***374*** | ***357,15*** | 9,68 | 5,33 | 124,36 | 9,91 | -16,85 | 0 | 3,96 |
|  | pectoral profundus 1 | -77,63 | 23,15 | -169,44 | -23,8 | -0,34 | 156,4 | ***-21,8*** | ***-0,34*** | ***373,13*** | 3,05 |
|  | pectoral profundus 2 | -98,14 | -14,52 | -143,5 | -23,8 | -0,34 | 156,4 | ***-21,8*** | ***-0,34*** | ***373,13*** | 3,05 |
|  | pectoral profundus 3 | -114,54 | -48,8 | -119,43 | -23,8 | -0,34 | 156,4 | ***-21,8*** | ***-0,34*** | ***373,13*** | 3,05 |
|  | pectoral profundus 4 | -128,17 | -82,59 | -98,18 | -23,8 | -0,34 | 156,4 | ***-21,8*** | ***-0,34*** | ***373,13*** | 3,05 |
|  | pectoral superficialis (descendens) | -67,02 | 49,63 | -192,14 | 2,12 | 14,69 | 95,96 | ***4,12*** | ***14,69*** | ***312,69*** | 1,17 |
|  | pectoral superficialis (transverse) | -77,13 | 32,16 | -177,87 | 2,12 | 14,69 | 95,96 | ***4,12*** | ***14,69*** | ***312,69*** | 4,74 |
|  | triceps medialis | ***-9,91*** | ***374*** | ***357,15*** | -11,93 | 7,15 | 118,98 | 9,91 | -16,85 | 0 | 1,73 |
| Antebrachium stabilizers | supinator | ***2,39*** | ***333,81*** | ***155,4*** | 13,45 | 4,98 | -11,42 | -2,39 | 17,08 | 0 | 0,38 |
|  | pronator terres | ***8,48*** | ***333,07*** | ***155,4*** | -19,95 | 0,82 | -1,09 | -8,48 | 16,34 | 0 | 0,36 |
|  | ulnaris lateralis | ***-14,24*** | ***321,39*** | ***155,4*** | 12,76 | -7,32 | -8,09 | 14,24 | 4,66 | 0 | 0,42 |
|  | tensor fascia antebrachi | ***-7,16*** | ***313,73*** | ***155,4*** | 7,16 | -3 | -216,73 | 7,16 | -3 | 0 | 0,34 |
|  | extensor carpi radialis | ***10,28*** | ***324,31*** | ***155,4*** | 12,62 | -9,92 | 14,1 | -10,28 | 7,58 | 0 | 1,23 |
|  | common digitalis extensor | ***15,45*** | ***343,69*** | ***155,4*** | 18,58 | 3,47 | 1,63 | -15,45 | 26,96 | 0 | 0,60 |
|  | lateralis digital extensor | ***3,96*** | ***345,32*** | ***155,4*** | 15,97 | 0,50 | -8,50 | -3,96 | 28,59 | 0 | 0,25 |
|  | flexor carpi radialis | ***4,65*** | ***313,6*** | ***155,4*** | -18,35 | -5 | -5,32 | -4,65 | -3,13 | 0 | 0,77 |
|  | superficial digital flexor | ***10,79*** | ***333,62*** | ***155,4*** | -14,1 | -11,52 | -9,19 | -10,79 | 16,89 | 0 | 0,74 |
| Flexors | latissimus dorsi (thoracis) | -31,5 | -64,08 | 58,97 | -10,04 | 10,88 | 119,11 | - | - | - | 2,82 |
|  | latissimus dorsi (lumbar) | -41,3 | -118,84 | 166,99 | -10,04 | 10,88 | 119,11 | - | - | - | 2,17 |
|  | teres major | -2,57 | -43 | -39,84 | -10,04 | 10,88 | 119,11 | - | - | - | 2,05 |
|  | teres minor | -6,64 | -11 | -129,82 | 6,90 | -9,45 | 150,63 | - | - | - | 0,71 |
|  | infraspinatus | 1,4 | -6,80 | -70,68 | 7,24 | 0,99 | 161,55 | - | - | - | 3,63 |
| Extensors | supraspinatus | 4,47 | 21,18 | -68,18 | -0,37 | 8,32 | 168,2 | - | - | - | 4,46 |
|  | brachiocephalicus | 116,85 | 128,61 | 12,93 | -0,72 | 7,13 | 47,37 | - | - | - | 1,52 |
|  | coracobrachialis | -4,88 | 16,42 | -146,01 | -12,77 | 1,82 | 118,73 | - | - | - | 0,51 |
|  | biceps brachii | 2,7 | 19,53 | -147,07 | ***-13,62*** | ***4,6*** | ***-216,73*** | -13,62 | 8,6 | 0 | 2,04 |
| Abductors | deltoideus (scapular) | 24,69 | 3,38 | -56,95 | 11,84 | 9,82 | 104,45 | - | - | - | 1,79 |
|  | deltoideus (acromial) | 29,53 | 0,93 | -139,05 | 11,84 | 9,82 | 104,45 | - | - | - | 1,66 |
| Adductors | subscapularis | 5,49 | 3,1 | -81,13 | -28,72 | -4,00 | 160,54 | - | - | - | 3,85 |
